# Unraveling the Detoxification Mechanism of 2,4-Dichlorophenol by Marine-Derived Mesophotic Symbiotic Fungi Isolated from Marine Invertebrates

**DOI:** 10.3390/md17100564

**Published:** 2019-09-30

**Authors:** Efstratios Nikolaivits, Andreas Agrafiotis, Aikaterini Termentzi, Kyriaki Machera, Géraldine Le Goff, Pedro Álvarez, Suchana Chavanich, Yehuda Benayahu, Jamal Ouazzani, Nikolas Fokialakis, Evangelos Topakas

**Affiliations:** 1 Industrial Biotechnology & Biocatalysis Group, Biotechnology Laboratory, School of Chemical Engineering, National Technical University of Athens, 15780 Athens, Greece; snikolai@central.ntua.gr (E.N.); agrafiotisandreas.vep@gmail.com (A.A.); 2 Department of Pesticides Control and Phytopharmacy, Benaki Phytopathological Institute, 14561 Kifissia, Greece; a.termentzi@bpi.gr (A.T.); k.machera@bpi.gr (K.M.); 3 Institut de Chimie des Substances Naturelles, ICSN, Centre National de la Recherche Scientifique CNRS, 91198 Gif sur Yvette, Francejamal.ouazzani@cnrs.fr (J.O.); 4 iMare Natural S.L., 18600 Granada, Spain; 5 Faculty of Science, Chulalongkorn University, Bangkok 10330, Thailand; suchana.c@chula.ac.th; 6 Department of Zoology, Faculty of Life Sciences, Tel Aviv University, Tel Aviv 69978, Israel; yehudab@tauex.tau.ac.il; 7 Division of Pharmacognosy and Chemistry of Natural Products, Department of Pharmacy, University of Athens, 15771 Athens, Greece; fokialakis@pharm.uoa.gr

**Keywords:** 2,4-dichlorophenol, bioremediation, marine-derived fungi, invertebrate symbionts, mesophotic zone, metabolite analysis, HRMS/MS

## Abstract

Chlorophenols (CPs) are environmental pollutants that are produced through various anthropogenic activities and introduced in the environment. Living organisms, including humans, are exposed to these toxic xenobiotics and suffer from adverse health effects. More specifically, 2,4-dichlorophenol (2,4-DCP) is released in high amounts in the environment and has been listed as a priority pollutant by the US Environmental Protection Agency. Bioremediation has been proposed as a sustainable alternative to conventional remediation methods for the detoxification of phenolic compounds. In this work, we studied the potential of fungal strains isolated as symbionts of marine invertebrates from the underexplored mesophotic coral ecosystems. Hence, the unspecific metabolic pathways of these fungal strains are being explored in the present study, using the powerful analytical capabilities of a UHPLC-HRMS/MS. The newly identified 2,4-DCP metabolites add significantly to the knowledge of the transformation of such pollutants by fungi, since such reports are scarce.

## 1. Introduction

In everyday life, we come across over 60,000 chemicals in the types of consumer products, drugs, pesticides, food additives, fuels, and industrial solvents [1]. Chlorophenols (CPs) constitute a class of organic compounds containing at least one chlorine atom attached on a phenol moiety. CPs are introduced in the environment as metabolites of herbicides and other chlorinated xenobiotics or through anthropogenic activities as effluent discharge of industrial processes; for instance, pulp bleaching, dye manufacturing, water disinfection with chlorine, waste burning, and wood waste incineration [2,3].

These compounds can be detected in water, soil and the atmosphere after volatilization and they can also accumulate in the adipose tissue of living organisms due to their lipophilicity [2,3]. 2,4-Dichlorophenol (2,4-DCP) has been widely used as a fungicide, pesticide, and wood preservative [4], being released in high amounts into the environment (ca. 10,000 kg in the US during 2014) according to the United States Environmental Protection Agency (US EPA), who listed it as a priority pollutant among other CPs [2,5]. Human exposure to CPs takes places by consuming substances that contain them or through skin [6]. CPs are toxic, endocrine disrupting substances that have been associated with a variety of adverse health effects such as oxidative stress, cytotoxicity, mutagenicity, carcinogenicity, and apoptosis induction [2,6,7].

Several physicochemical methods have been used so far for the remediation of phenolic compounds including photocatalysis, ozonation, liquid–liquid or solid-state extraction, adsorption, ion-exchange, membrane-based separation, electro-Fenton reaction, mixing coagulation and supersonic chemistry, many of which suffer from several disadvantages such as high operational cost and incomplete degradation [8,9].

Bioremediation is the use of living organisms in order to remove pollutants from soil and water; a method that is considered more cost-effective and environmentally friendly than the conventional techniques mentioned above [5,8]. Microorganisms—mainly bacteria and fungi—indigenous to the contaminated regions are potential candidates for the task, benefiting from their acquired enzymatic arsenal, aiming to use the contaminants as food, ideally towards their complete mineralization [10]. Fungi are robust organisms and most of them are usually more tolerant to high concentrations of pollutants compared to bacteria [10].

The marine environment is an untapped source of microbial diversity, showing various characteristics valuable for biotechnological applications, including bioremediation [11], especially considering that a great part of the Earth’s pollution appears in the oceans. An exceptionally under-investigated source of marine biodiversity is the fungal symbionts of marine invertebrates (e.g., ascidians, cnidarians, and sponges) [11] and more specifically the ones residing in the mesophotic zone. Mesophotic coral ecosystems (MCEs) are tropical or sub-tropical light-dependent habitats located at 30–40 m below the sea level and extend as deep as 150 m. Even though they are known for the rich biodiversity, they remain relatively under investigated, due to the limitations imposed by their location. Only recently, they have gained more attention as a result of the technological advances, such as remotely operated vehicles (ROVs) and autonomous underwater vehicles (AUVs) [12].

Microorganisms derived from pristine environments do not necessarily possess the ability to detoxify halogenated organic pollutants, as they are unlikely to have ever come across such molecules in order to evolve special detoxification mechanisms. However, there are natural halogenated compounds found in the sea [13], some of them similar to manmade pollutants, like brominated diphenyl ethers. Many sessile organisms, such as sponges and corals (or their symbionts), are thought to produce organohalogens as a defense against predators [14]. Therefore, marine-derived invertebrate symbionts may have the potential to bioconvert halogenated aromatic compounds.

The present work describes the screening of 60 fungal strains isolated from pristine MCEs habitats as symbionts of invertebrates for their potential in bioremediation of 2,4-DCP. The strains that showed the highest 2,4-DCP bioconversion yield were chosen for further investigation of their metabolic pathways.

## 2. Results and Discussion

Mesophotic coral ecosystems have been rather neglected by scientists, compared to the shallow reefs, mostly because of technical challenges. The data we have obtained about the biodiversity of these ecosystems are rather biased due to the limited geographical regions investigated so far, namely the Western Atlantic Ocean, the Hawaiian Archipelago, the Great Barrier Reef (Australia), and the Red Sea [15]. In this work, we tried to access the biodiversity of symbiotic fungal strains isolated from mesophotic invertebrates and their potential in bioremediation of 2,4-DCP. The fungi were isolated from invertebrates collected in four different locations, namely Andaman Sea, Eilat (northern Red Sea), West and East Mediterranean Sea. The invertebrates were corals and sponges (see Table 1).

Among the 60 isolates screened, 52 have the capability to convert significantly 2,4-DCP (Table 1). 81% of them belong to three genera—*Aspergillus* (31%), *Penicillium* (33%), and *Cladosporium* (17%). Twenty isolates convert 2,4-DCP over 40% at 1 mM initial concentration, including 7 *Aspergillus,* 8 *Penicillium,* 2 *Cladosporium,* 1 *Obolarina,* 1 *Pseudocercosporella*, and 1 *Chrysosporium* species. However, the capacity of conversion could not be linked to the area of collection, the depth nor the nature of the host invertebrate, indicating that only the genetic background and metabolic capabilities of specific genera is required and is slightly impacted by environmental habitat condition.

Marine-derived fungi are promising microorganisms to be utilized for biotechnological applications, thanks to unique properties they acquired through their adaptation to extreme environmental conditions, such as high salinity, low oxygen concentration, high pressure, temperature, and low nutrient availability [11,16]. So far, marine-derived fungi have been employed for the bioremediation of crude oil components, like polycyclic aromatic hydrocarbons (PAHs— phenanthrene, pyrene, benzo[a]pyrene) and aliphatic alkenes, but also 1,4,6-trinitrotoluene and hexahydro-1,3,5-trinitro-1,3,5-triazine, originating from unexploded ordnance [16,17,18]. There are also some reports regarding the degradation of pesticides—most of them chlorinated compounds—like 1,1-dichloro-2,2-bis-(4-chlorophenyl)ethane (DDD), esfenvalerate, dieldrin, and methyl parathion [16].

### 2.1. 2,4-DCP Biotransformation Potential of Isolated Fungi

In the present work, the fungal isolates were tested for their ability to transform the environmental pollutant 2,4-DCP in resting-cell reactions. The transformation yield of 2,4-DCP for each of the 52 strains at the 10th reaction day is presented in Table 1. Only 9 microorganisms were able to reach over 50% bioconversion yield. Out of these, five could bioconvert the pollutant at percentages over 55%, namely *Penicillium steckii* TM2-S5 (58.5%), *Chrysosporium* sp.TM9-S2 (74.0%), *Penicillium* sp. TM38-S1 (56.2%), *Aspergillus creber* TM122-S3 (62.0%), and *Aspergillus* sp. TM124-S1 (69.0%). It should be noted that the initial concentration of 2,4-DCP was high (1 mM or 163 mg L^−1^), therefore, only the strains with increased tolerance on this pollutant and high biotransformation potential could achieve increased yields.

Vroumsia et al. [19] screened 90 fungal strains able to detoxify other xenobiotics (phenylurea herbicides, pentachloronitrobenzene, and pentachlorophenol) for their ability to biodegrade 2,4-DCP at initial concentration of 100 mg L^−1^. Out of these, only three were able to reach yields above 50%, after 5 d. A *Bacillus* strain that was isolated from chlorophenol enrichment cultures of a paper mill aeration pond could remove ~25% of 0.56 mM 2,4-DCP after 9 d [20]. A *Pseudomonas putida* strain not related to chlorophenols was able to remove 35% from 51 mg L^−1^ 2,4-DCP [21]. In the same manner, Basidiomycete *Phanerochaete chrysosporium* could bioconvert ~20% of 100 mg L^−1^ 2,4-DCP in the presence of 2 mg L^−1^ cadmium. For initial concentrations higher than 40 mg L^−1^ 2,4-DCP the total removal was less than 40% after 4.5 d [22].

### 2.2. Identification of 2,4-DCP Metabolites

When it comes to the biotransformation of CPs, the majority of studies use bacteria, while reports with fungi (let alone marine-derived) are very scarce. Bacteria of various genera have the ability to aerobically degrade all kinds of CPs. More specifically, representatives of the genera *Pseudomonas*, *Rhodococcus*, *Bacillus*, and *Cupriavidus* have the ability to degrade DCP [6]. The general mechanism for the bacterial aerobic degradation involves the initial *ortho*-hydroxylation of DCP, followed by the *ortho*-cleavage of the catechol scaffold and simultaneous release of the *para* chloride leading to 2-chloromaleylacetic acid, which is further dehalogenated [3]. *Cupriavidus necator* JMP222 is reported to degrade 2,4-DCP through a *meta*-cleavage pathway, yielding 2-hydroxy-3,5-dichloro-6-oxo-hexa-2,4-dienoic acid [6].

The detoxification of xenobiotics by higher organisms and fungi may take place in two stages known as phase I and phase II. Phase I involves the modification of the compound by the addition of a functional group, usually performed by cytochrome P450 enzymes. The products of phase I may then undergo conjugation reactions to form sulfates, glucuronides, glucosides, or glutathione conjugates. The phase II conjugation enzymes (sulfotransferases, glycosyltransferases, glutathione S-transferases) are non-specific, creating less-toxic metabolites that can be easily excreted from the cell and are typically not subjected to further modifications [23,24].

The increased resolving power of 70,000 at the full scan and of 35,000 of the MS/MS measurements in correlation with the accurate mass measurements (Δm < 5 ppm), of the Orbitrap analyzer allowed the safe characterization of several compounds in the reaction mixtures. Out of the characterized components, special attention was given to those indicated by the 2,4-DCP metabolism study, as possible metabolites and degradation products. Extended identification of the xenobiotic metabolites was performed by the Compound Discoverer 2.1 software (Thermo Fisher Scientific^TM^ - San Jose, CA, USA) in comparison with internal spectral libraries. Those indicated compounds were further evaluated in comparison with data from literature and were finally indicated as intermediates or final products of the DCP metabolism in the presence of specific microbial strain. The highlighted compounds, described below, were not present in control samples. The suggested elemental composition (EC), the indicated ring double bond equivalents (RDBeq) and the special isotopic patterns for the chlorinated and sulphated molecules not only for the molecular ion, but also for the HRMS/MS fragment, helped significantly with the structural identification procedure. 

The detected compounds indicated as 2,4-DCP metabolites, based on the above-mentioned identification procedure are presented in Table 2.

Out of the five strains tested, *Chrysosporium* sp. ΤΜ9-S2 was the richest in metabolites, containing all of the compounds mentioned in Table 2 except two, while *Penicillium* sp. TM38-S1 was the poorest in metabolites (only two detected). Overall, the only ‘Phase I’ metabolites identified were dichlorocatechol (or some kind of hydroxylated dichlorophenol) (**11**) and 2-chlorohydroquinone (or 4-chlorocatechol) (**7**) that were only present in *Chrysosporium* sp. ΤΜ9-S2. Dichlorocatechol was further metabolized to its corresponding palmitate (**4**), glucoside (**8**), and glutamine conjugates (**2**). Chlorohydroquinone was further conjugated with glutamine (**1**). *Chrysosporium* sp. ΤΜ9-S2 was able to transform 2,4-DCP to its cysteine conjugate (**6**), while also *P. steckii* TM2-S5 and *A. creber* TM122-S3 could reductively dechlorinate the initial compound to form chlorophenol (**10**). Chlorophenol was further transformed by *Chrysosporium* sp. ΤΜ9-S2 to its cysteine conjugates (**5**), when *P. steckii* TM2-S5, *A. creber* TM122-S3, and *Aspergillus* sp. TM124-S1 produced its sulphated metabolite (**9**). A fact that should be noted is that strains *A. creber* TM122-S3 and *Aspergillus* sp. TM124-S1 could completely oxidatively dechlorinate the initial compound to form hydroxyquinol (**3**), which could possibly be further assimilated by these strains.

### 2.3. Fungal Mechanisms for 2,4-DCP Detoxification

The mechanisms by which fungi deal with 2,4-DCP are much less studied compared to bacteria and also seem to be much more complicated and diverse. To our knowledge, this is the first report of several DCP metabolites mentioned above. The soil fungus *Mortierella* sp. could bioconvert DCP using two different pathways: the one involved the initial hydroxylation to dichlorocatechol and further methylation to dichloroguaiacol, while the second included the dechlorination of the *para*-chlorine yielding 2-chloro-hydroquinone (as in *Chrysosporium* sp. ΤΜ9-S2) and the subsequent dechlorination leading to hydroquinone [25]. *Cunninghamella elegans* expressed both Phase I and Phase II enzymatic activities for the bioconversion of xenobiotics including hydroxylase and demethylase, as well as glucuronosyl-, glycosyl-, and sulfotransferases. This fungus could also dechlorinate 1-chloro-2,4-dinitrobenzene by the use of a glutathione S-transferase [26]. The same fungus could use both oxidative and reductive mechanisms for the biotransformation of substituted naphthalene [27]. Apparently, a combination of oxidative and reductive dechlorination can take place in aerobic microorganisms like in the case of *Flavobacterium* sp., which could bioconvert pentachlorophenol firstly to tetrachlorohydroquinone by oxidative dechlorination and then have two more chlorine atoms removed by reductive dehalogenation [28]. The reductive dehalogenase of this bacterium that utilized glutathione as a reducing agent, was isolated and studied and it appeared to be similar to plant glutathione S-transferases [29,30]. Additionally, a marine-derived *Trichoderma harzianum* strain isolated from ascidian *Didemnun ligulum* could biotransform pentachlorophenol and produce pentachloroanisole and 2,3,4,6-tetrachloroanisole suggesting a reductive dehalogenation activity [31]. The white-rot fungus *Coriolus versicolor* followed different pathways for the transformation of the herbicide chloronitrofen—including hydroxylations, oxidative, and reductive dechlorination reactions [32].

Many other fungi, mostly white-rot, have the ability to transform DCP and other CPs by the powerful ligninolytic enzymatic activities expressed extracellularly. The products of these activities could be even more toxic than the initial CPs, like in case of chlorinated ethers and dioxins [33,34]. White-rot fungi *Panus tigrinus* and *Coriolus versicolor* showed the ability to bioconvert various CPs including DCP and 2,4,6-trichlorophenol (TCP) by inducing ligninolytic activities [35]. The TCP fungal metabolites were identified as 2,6-dichloro-1,4-hydroquinol (oxidative dechlorination) and 2,6-dichloro-1,4-benzoquinone. *Phanerochaete chrysosporium* is a well-known ligninolytic white-rot mushroom with the ability to transform DCP. All the metabolites identified from this strain were previously reported and include the halogenated compounds 2,4-dichloroanisole, 2-chloro-1,4-hydroquinone (present study), 2-chloro-1,4-benzoquinone, and 2-chloro-1,4-dimethoxybenzene [36]. Furthermore, several completely dechlorinated metabolites were detected, such as 2,5-dimethoxy-1,4-hydroquinone, 2,5-dimethoxy-1,4-benzoquinone, 2,5-dihydroxy-1,4-benzoquinone, and 1,2,4,5-tetrahydroxybenzene. Stoilova et al. [37] studied the removal of high concentrations of DCP by *Aspergillus awamori* NRRL 3112 and even though no metabolites were identified, catechol 1,2-dioxygenase activity was detected suggesting that the degradation pathway is different than the ones described above. In our case, the catechol dioxygenase activities measured for all tested strains were very low, estimated less than 8 U mg^−1^ of expressed extracellular protein (data not shown).

**Figure 1:**
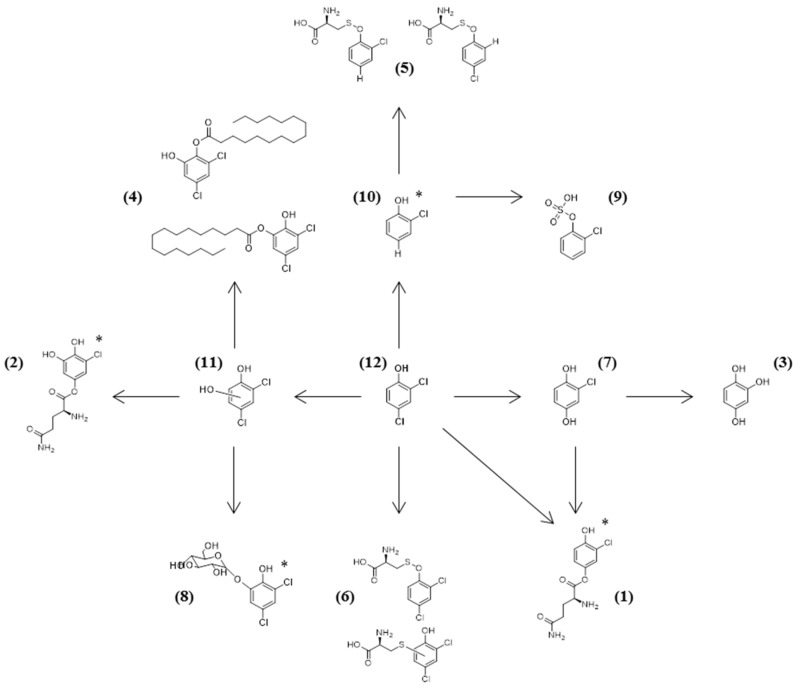
Proposed metabolic pathway for the detoxification of 2,4-DCP by the isolated mesophotic fungi. The isomers were suggested according to MS^2^ data and literature. For metabolites where there is no information about the most probable isomer, an asterisk has been added next to the molecule. The number next to each compound is the one corresponding to Table 2.

Based on the above-mentioned literature and our identification methodology, we tried to create a schematic representation of the 2,4-DCP metabolic pathway by the tested fungal strains. Structural configurations presented in Figure 1 are tentative and are based on the most possible conformation according to the compound dynamics and data from literature. In the case of the compounds that are represented by two peaks with close retention times in the chromatograms, we assumed that more than one isomer is present.

## 3. Materials and Methods 

### 3.1. Chemicals

2,4-DCP (99%) was purchased from Sigma-Aldrich (St. Louis, MO, USA). Organic solvents (acetonitrile and chloroform) were of HPLC grade (Fisher Chemical, Pittsburgh, PA, USA). LC-MS grade acetonitrile and formic acid were purchased from Fisher Scientific (Fisher Optima, Loughborough, UK) and LC-MS water was produced from SG Millipore apparatus.

### 3.2. Culture Conditions and Resting-Cell Reactions

Fungal strains were grown on Difco™ Marine Agar 2216 (BD Biosciences, San Jose, CA, USA) plates at 27 °C for 5 days. These plates were used to inoculate submerged cultures with Difco™ Marine Broth 2216 (pH 7.6) at 27 °C and 150 rpm shacking. After 5 days, the biomass was filtered using 0.2 μm-pore filters and used as a biocatalyst (10% *w*/*v*) in 15-mL reactions containing 1 mM DCP in ultrapure water. Reactions were left under mild shacking at 27 °C for 10 days. Samples were withdrawn on the third and sixth day and analyzed after filtration. On the final day, the remaining reaction was extracted with equal volume of chloroform and it was analyzed after drying and recovery in ultrapure water.

### 3.3. Identification of Fungal Strains

Genomic DNA of the strains were isolated using DNeasy Plant Mini Kit (Qiagen, Germantown, MD, USA) according to manufacturer’s instructions. For each sample, the ITS rDNA region was amplified with primers ITS1F (5′-CTTGGTCATTTAGAGGAAGTAA-3′) and ITS4 (5′-TCCTCCGCTTATTGATATGC). Amplicons were sequenced by Sanger sequencing (Eurofins genomics, GATC Biotech, Konstanz, Germany) and the sequences were submitted to non-redundant database of the NCBI using BLASTn program (GenBank) and compared to the corresponding sequences.

### 3.4. Culture Conditions and Resting-Cell Reactions

Reaction samples were analyzed for the quantification of the remaining 2,4-DCP using a SHIMADZU LC-20AD HPLC equipped with a SIL-20A autosampler. A C-18 reverse-phase NUCLEOSIL® 100-5 (Macherey-Nagel, Düren, Germany) served as the stationary phase and 40% aqueous acetonitrile as the mobile phase (flow rate 0.8 mL min^−1^). Detection took place with the photodiode array detector Varian ProStar and the wavelength at which the absorption was recorded was 285 nm. The total running time was 16 min and the retention time of DCP was 12.4 min.

### 3.5. Identification of 2,4-DCP Metabolites by UHPLC-HRMS/MS

The analysis of the 2,4-DCP metabolites was performed by UHPLC-HRMS/MS spectrometry on a Q-Exactive Orbitrap platform (Thermo Fisher Scientific, San Jose, CA, USA). The ultra-high performance liquid chromatography was performed employing a Dionex Ultimate 3000 UHPLC system (Thermo Scientific™ Dionex™, Sunnyvale, CA, USA) equipped with a binary pump, an autosampler, an online vacuum degasser, and a temperature-controlled column compartment. A Hypersil Gold UPLC C18 (2.1 × 150 mm, 1.9 μm) reversed phased column (Thermo Fisher Scientific, San Jose, CA, USA). was used for the analysis. The gradient phase consisted of solvents A: aqueous 0.1% (*v*/*v*) formic acid and B: acetonitrile. The gradient elution was: T = 0 min, 5% B; T = 2 min, 5% B, T = 23 min, 95% B, T = 28 min, 95% B, T = 28.1 min, 5% B; T = 30 min, 5% B. The flow rate was 0.220 mL/min and the injection volume was 3 μL. The column temperature was kept at 40 °C while the sample tray temperature was set at 10 °C.

The high-resolution mass spectrometry was performed on an Orbitrap Q-Exactive mass spectrometer (Thermo Fisher Scientific, San Jose, CA, USA). The ionization was performed at HESI, both positive and negative modes. The conditions for the HRMS for both negative and positive ionization modes were set as follows: capillary temperature, 350 °C; spray voltage, 2.7 kV; S-lense Rf level, 50 V; sheath gas flow, 40 arb. units; aux gas flow, 5 arb. units; aux. gas heater Temperature, 50 °C. For the full scan experiments the resolution was 70,000. The data dependent acquisition capability has been also used at 35,000 resolution, allowing for MS/MS fragmentation of the three most intense ions of every peak exceeding the predefined threshold applying a 10 s dynamic exclusion. Stepped normalized collision energy was set at 40, 60, and 100. The acquisition of the mass spectra was performed in every case using the centroid mode. Data acquisition and analysis has been completed employing Xcalibur 2 (Thermo Fisher Scientific, Bremen, Germany).

Data were imported to Compound Discoverer 2.1 (Thermo Fisher Scientific, San Jose, CA, USA) and a standard metabolism workflow was selected in order to detect the possible DCP metabolites in the extracts. The appropriate set up for peak detection, deconvolution, deisotoping, and alignment was applied.

Results revealed several 2,4-DCP metabolites in resting cell reactions compared to control samples (just cells).

## 4. Conclusions

In the present work, we aimed in the investigation of the potential of marine-derived fungi for the detoxification of the 2,4-DCP. In order to accomplish that, we accessed the underexplored mesophotic zone, collecting marine invertebrates from different regions. The fungal symbionts of these invertebrates were isolated, identified, and used as whole-cell biocatalysts for the removal of 2,4-DCP. Even though these strains originate from non-polluted habitats, they showed high biotransformation potential. In an attempt to elucidate the mechanism by which these marine-derived microorganisms biotransform 2,4-DCP, we detected their metabolites using UHPLC-HRMS/MS analysis. Based on these results and the literature, we saw that the isolates use non-specific enzymatic activities in order to biotransform the xenobiotic and convert it to less toxic products.

## Figures and Tables

**Table 1:** Percentage of 2,4-DCP reduction in resting-cell reactions after 10 d for all isolated fungal strains, which were identified based on their ITS region. Information (region and depth) about the invertebrate host of each strain are given. Locations: East Mediterranean Sea (Med E), West Mediterranean Sea (Med W), northern Red Sea (Eilat), and Andaman Sea.

Isolate Code	Invertebrate of Origin	Location	Depth (m)	Isolate Identification	% DCP Removal
TM2-S5	Sponge on top of *Spondylus*	Med E	38	*Penicillium steckii*	58.5
TM6-S1	*Dendronephthya* sp.	Eilat	120	*Aspergillus tubingensis*	23.2
TM6-S2	*Dendronephthya* sp.	Eilat	120	*Penicillium kewense*	9.3
TM37-S2	*Spondylus* sp.	Eilat	49	*Penicillium chrysogenum*	49.2
TM38-S1	*Polycarpa* sp.	Eilat	49	*Penicillium* sp.	56.2
TM9-S2	*Theonella* sp.	Eilat	65	*Chrysosporium* sp.	74.0
TM43-S1	*Xestospongia* sp.	Andaman	30–40	*Penicillium* sp.	17.5
TM43-S3	*Xestospongia* sp.	Andaman	30–40	*Cladosporium halotolerans*	4.5
TM46-S1	*Thrinacophora* sp.	Andaman	30–40	*Cladosporium* sp.	30.4
TM47-S1	*Chondrilla* sp.	Andaman	30–40	*Cladosporium halotolerans*	46.9
TM116-S2	*Xestospongia* sp.	Andaman	30–40	*Aspergillus* sp.	28.4
TM116-S3	*Xestospongia* sp.	Andaman	30–40	*Hortaea* sp. (yeast)	9.5
TM122-S1	*Iotrochota* sp.	Andaman	30–40	*Aspergillus* sp.	21.7
TM122-S2	*Iotrochota* sp.	Andaman	30–40	*Aspergillus fumigatus*	29.5
TM124-S1	*Echinaster sepositus*	Med W	30–40	*Aspergillus* sp.	69.0
TM125-S2	*Clavelina dellavallei*	Med W	30–40	*Penicillium* sp.	51.6
TM125-S3	*Clavelina dellavallei*	Med W	30–40	*Penicillium* sp.	50.2
TM126-S1	*Clayx nicaensis*	Med W	30–40	*Aspergillus* sp.	47.0
TM133-S2	*Cerianthus membranaceus*	Med W	30–40	*Penicillium crustosum*	10.6
TM138-S1	*Didemnum maculosum*	Med W	30–40	*Purpureocillium lilacinum*	26.2
TM138-S3	*Didemnum maculosum*	Med W	30–40	*Cladosporium* sp.	16.8
TM138-S4	*Didemnum maculosum*	Med W	30–40	*Purpureocillium lilacinum*	24.6
TM2-S6	Sponge on top of *Spondylus*	Med E	38	*Aspergillus* sp.	55.6
TM7-S1	*Siphonogorgia* sp.	Eilat	120	*Penicillium* sp.	29.3
TM30-S1	*Diacarnus erythraenus*	Eilat	49	*Penicillium fellutanum*	37.1
TM220-S1	Gorgonian overgrown by *Antipathozoanthus* sp.	Eilat	75	*Cladosporium sphaerospermum*	40.9
TM220-S4	Gorgonian overgrown by *Antipathozoanthus* sp.	Eilat	75	*Penicillium* sp.	47.1
TM225-S2	*Villogorgia nozzolea*	Eilat	68	*Penicillium chrysogenum*	9.0
TM226-S1	*Callyspongia* sp.	Eilat	86	*Cladosporium halotolerans*	36.2
TM226-S2	*Callyspongia* sp.	Eilat	86	*Penicillium chrysogenum*	9.7
TM230-S1	*Siphonogorgia* sp.	Eilat	115	*Penicillium* sp.	55.0
TM230-S6	*Siphonogorgia* sp.	Eilat	115	*Penicillium* sp.	32.5
TM242-S1	Orange/beige gorgonian	Eilat	152	*Penicillium chrysogenum*	44.0
TM53-S1	*Plakina* sp.	Andaman	30–40	*Cladosporium* sp.	16.3
TM54-S1	*Terpios* sp.	Andaman	30–40	*Aspergillus* sp.	40.0
TM58-S1	*Biemna* sp.	Andaman	30–40	*Penicillium* sp.	30.0
TM58-S2	*Biemna* sp.	Andaman	30–40	*Aspergillus niger*	46.0
TM65-S2	*Padina* sp	Andaman	30–40	*Pseudocercosporella* sp.	46.4
TM65-S4	*Padina* sp	Andaman	30–40	*Acremonium* sp.	28.3
TM75-S1	*Ulva* sp.	Andaman	30–40	*Aspergillus* sp.	21.6
TM83-S1	Bryozoan	Andaman	30–40	*Cladosporium halotolerans*	15.6
TM83-S2	Bryozoan	Andaman	30–40	*Cladosporium halotolerans*	33.7
TM116-S1	*Xestospongia* sp.	Andaman	30–40	*Aspergillus aculeolatus*	24.6
TM122-S3	*Iotrochota* sp.	Andaman	30–40	*Aspergillus creber*	62.0
TM122-S4	*Iotrochota* sp.	Andaman	30–40	*Obolarina* sp.	40.1
TM124-S4	*Echinaster sepositus*	Med W	30–40	*Aspergillus* sp.	36.0
TM124-S7	*Echinaster sepositus*	Med W	30–40	*Aspergillus* sp.	46.4
TM132-S1	*Haliclona mediterranea*	Med W	30–40	*Aspergillus* sp.	24.9
TM141-S1	*Cliona celata*	Med W	30–40	*Alternaria* sp.	22.3
TM141-S2	*Cliona celata*	Med W	30–40	*Alternaria* sp.	16.3
TM141-S3	*Cliona celata*	Med W	30–40	*Alternaria* sp.	39.8
TM148-S1	*Astropecten bispinosus*	Med W	30–40	*Aspergillus* sp.	24.9

**Table 2:** 2,4-DCP metabolites traced only in DCP-treated cell cultures

A/A	Rt (Min)	[M − H]^−^	EC	MS^2^	Found in
(1)	4.90	271.0480	C_11_H_13_ClN_2_O_4_	114.9515 (C_4_O_2_Cl), 191.0924 (C_11_H_13_O_2_N)	*Penicillium steckii* TM2-S5, *Chrysosporium* sp. ΤΜ9-S2, *Aspergillus creber* TM122-S3, *Aspergillus* sp. TM124-S1
(2)	6.98	287.0429	C_11_H_13_O_5_N_2_Cl	-	*Chrysosporium* sp. ΤΜ9-S2, *Aspergillus* sp. TM124-S1, *Penicilluim* sp. TM38-S1
(3)	7.01	125.0233	C_6_H_6_O_3_	-	*Penicillium steckii* TM2-S5, *Penicilluim* sp.TM38-S1, *Aspergillus creber* TM122-S3, *Aspergillus* sp. TM124-S1
(4)	8.87	415.1801	C_22_H_34_Cl_2_O_3_	-	All
(4)	9.06	415.1801	C_22_H_34_Cl_2_O_3_	-	All
(5)	9.42	245.9986	C_9_H_10_NClO3S	158.9675 (C_6_H_4_OClS)	*Chrysosporium* sp. ΤΜ9-S2
(5)	9.63	245.9986	C_9_H_10_NClO3S	158.9675 (C_6_H_4_OClS)	*Chrysosporium* sp. ΤΜ9-S2
					
(6)	10.24	279.9596	C_9_H_9_O_3_NSCl_2_	-	*Chrysosporium* sp. ΤΜ9-S2
(6)	10.42	279.9596	C_9_H_9_O_3_NSCl_2_	-	*Chrysosporium* sp. ΤΜ9-S2
(7)	11.68	142.9903	C_6_H_5_ClO_2_	114.9515 (C_4_O_2_Cl)	*Chrysosporium* sp. ΤΜ9-S2
(8)	12.33	339.0033	C_12_H_14_Cl_2_O_7_	160.9565 (C_6_H_3_OCl_2_), 124.9798 (C_6_H_2_OCl)	*Chrysosporium* sp. ΤΜ9-S2
(9)	12.43	206.9513	C_6_H_5_O_4_ClS	-	*Penicillium steckii* TM2-S5, *Aspergillus creber* TM122-S3, *Aspergillus* sp. TM124-S1
(10)	13.37	126.9945	C_6_H_5_OCl	-	*Penicillium steckii* TM2-S5, *Chrysosporium* sp. ΤΜ9-S2, *Aspergillus creber* TM122-S3
(11)	13.81	176.9505	C_6_H_4_Cl_2_O_2_	-	*Chrysosporium* sp. ΤΜ9-S2
(11)	13.94	176.9505	C_6_H_4_Cl_2_O_2_	-	*Chrysosporium* sp. ΤΜ9-S2
(12)	15.12	160.9565	C_6_H_4_Cl_2_O	124.9798 (C_6_H_2_OCl); 88.9943 (C_3_H_2_OCl)	All

Rt = retention time; [M − H]^−^= m/z of the pseudomolecular ion; EC = elemental composition; MS^2^ = the main MS/MS fragments for each pseudomolecular ion together with the respective EC.
